# The Systems Genetics Resource: A Web Application to Mine Global Data for Complex Disease Traits

**DOI:** 10.3389/fgene.2013.00084

**Published:** 2013-05-20

**Authors:** Atila van Nas, Calvin Pan, Leslie A. Ingram-Drake, Anatole Ghazalpour, Thomas A. Drake, Eric M. Sobel, Jeanette C. Papp, Aldons J. Lusis

**Affiliations:** ^1^Department of Human Genetics, University of CaliforniaLos Angeles, Los Angeles, CA, USA; ^2^Department of Pathology and Laboratory Medicine, University of CaliforniaLos Angeles, Los Angeles, CA, USA; ^3^Department of Medicine, University of CaliforniaLos Angeles, Los Angeles, CA, USA; ^4^Department of Microbiology, Immunology, and Molecular Genetics, University of CaliforniaLos Angeles, Los Angeles, CA, USA

**Keywords:** database, genomics, systems biology, data integration, web services, data analysis

## Abstract

The Systems Genetics Resource (SGR) (http://systems.genetics.ucla.edu) is a new open-access web application and database that contains genotypes and clinical and intermediate phenotypes from both human and mouse studies. The mouse data include studies using crosses between specific inbred strains and studies using the Hybrid Mouse Diversity Panel. SGR is designed to assist researchers studying genes and pathways contributing to complex disease traits, including obesity, diabetes, atherosclerosis, heart failure, osteoporosis, and lipoprotein metabolism. Over the next few years, we hope to add data relevant to deafness, addiction, hepatic steatosis, toxin responses, and vascular injury. The intermediate phenotypes include expression array data for a variety of tissues and cultured cells, metabolite levels, and protein levels. Pre-computed tables of genetic loci controlling intermediate and clinical phenotypes, as well as phenotype correlations, are accessed via a user-friendly web interface. The web site includes detailed protocols for all of the studies. Data from published studies are freely available; unpublished studies have restricted access during their embargo period.

## Introduction

**Figure 1:**
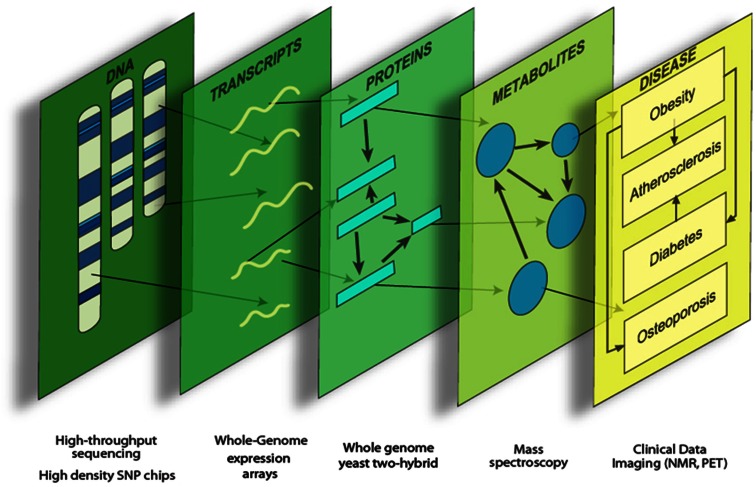
**Systems genetics analysis**. Systems genetics integrates genetic variation, intermediate phenotypes, and clinical traits to map loci and model biological networks underlying the intermediate phenotypes, which are subsequently related to the more complex clinical traits.

Systems genetics is an analysis of complex traits that integrates genetic variation, molecular traits (such as global transcript levels), and clinical traits. Data integration can provide a more comprehensive view of the various genetic functions and interactions underlying complex disease (Figure 1). Molecular traits can be viewed as “intermediate phenotypes,” since they are intermediate between DNA variation and final clinical traits. Generally, the intermediate phenotypes are determined using high throughput “omics” technologies, such as whole genome expression arrays, sequencing, and mass spectrometry (Flint and Mackay, 2009; Keller and Attie, 2010; MacLellan et al., 2012; Meng et al., 2013).

Systems genetics analyses generally involve the following steps: first, a population of individuals differing in the clinical traits of interest is identified. This population could be a group of unrelated individuals or a segregating population as in a family. Second, the individuals are examined for the clinical traits of interest. Third, the individuals are genotyped using high-density SNP arrays or simply a series of genetic markers. Fourth, the individuals are examined for intermediate phenotypes of interest such as global transcript levels, protein levels, or metabolite levels. Fifth, the data are integrated using results from genetic mapping studies (association or linkage), correlation between intermediate phenotypes and clinical traits, and network modeling. Sixth, based on these analyses, hypotheses about the relationships between DNA variation, intermediate phenotypes, and clinical traits are formulated. Seventh, hypotheses are validated using experimental perturbations, and the results used in an iterative fashion to refine the models.

To allow the genetics community to perform a systems genetics analysis for a broad array of traits, we are introducing a new, publicly available web application and database called the Systems Genetics Resource (SGR). The SGR is located at http://systems.genetics.ucla.edu and contains data from a number of mouse and human studies. The mouse data sets consist of several genetic crosses between inbred strains of mice as well as a population of mice termed the Hybrid Mouse Diversity Panel (HMDP). The human studies consist of several populations of individuals that have been genotyped with high-density SNP arrays and phenotyped for clinical traits and global transcript levels of particular cell types or tissues. The SGR includes studies on inflammation, cardiovascular traits, metabolism (including obesity and diabetes), and bone traits.

The web application provides easy access to pre-computed tables of genetic mapping results, correlation analyses between intermediate and clinical traits, and network modeling. No knowledge of how to use the underlying relational database is required. All study protocols are included. For published studies, all data are freely available. We are continuing to add new studies to this site, with their web access password-restricted until a primary publication or the end of an embargo period. We believe the ease and completeness of this site will encourage data exploration using the powerful systems genetics approach. Some of these data can also be accessed through an interactive web service for systems genetics^1^ or in the Mouse Phenome Project^2^.

## Motivation and Applications

**Figure 2:**
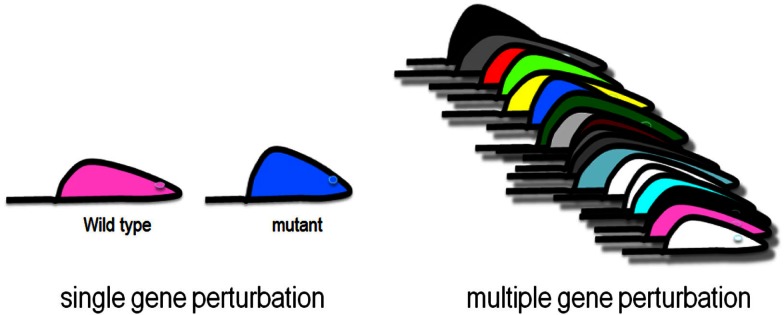
**Genetic analysis of complex traits**. Single gene perturbations such as Mendelian traits and knockout mouse models involve two states where causality is easy to establish. Collections of strains of mice, or randomly sampled people, are examples of multiple gene perturbations, which involve many states where interactions can be studied using correlation and network modeling.

**Figure 3:**
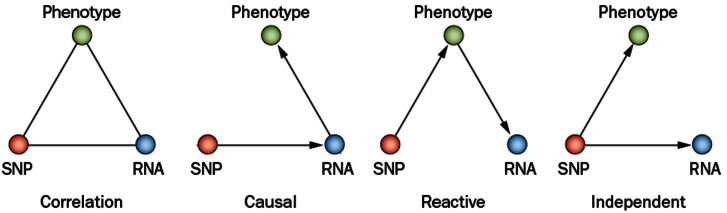
**Relationships between variation and correlated traits**. There are three possible causal relationships when there is correlation among a SNP (single nucleotide polymorphism), its transcript (RNA), and a physiological or pathologic trait (phenotype). In the causal model, the SNP variation affects its transcript levels leading to the resulting phenotype. In the reactive model, the SNP acts on the phenotypes, which in turn affects transcript. In the independent model, the SNP variation acts on both the phenotype and transcript independently (Schadt et al., 2005; Aten et al., 2008).

Complex genetic traits can be examined in two ways (Figure 2). The first is to carry out a series of single gene perturbations, such as the analysis of cases and controls for Mendelian traits or of genetically engineered mice. Such analyses provide results in which causality can be established in a straightforward manner. However, because in each perturbation there are only two states, wild-type and mutant, it is difficult to examine interactions, such as gene-by-gene interactions and gene-by-environment interactions. A second approach to studying complex traits is to examine individuals with multiple perturbations, such as the individuals in a population, which will differ at thousands or millions of genetic loci. In this approach, causality is more difficult to establish, but one can examine correlations between the elements of the systems (DNA variation, intermediate phenotypes, and clinical traits) using correlation, mapping, and network modeling. For example, suppose one is interested in studying the genetic factors and molecular pathways contributing to obesity. If we examine a population of individuals differing in obesity for transcript levels in relevant tissues, such as fat or hypothalamus, then we can correlate transcript levels with the clinical trait of adiposity or body mass index. The relationship between correlated genes and traits may be causal (that is, the genes may directly contribute to adiposity) or reactive (that is, the clinical trait may affect the intermediate phenotype), or the traits may be independent (see Figure 3), but in any case they provide hypotheses that can be tested. Similarly, if a genetic locus controls both transcript levels of a gene and body fat, this generates the hypothesis that the transcript may be involved in the clinical trait.

The SGR is intended primarily to assist biology-focused researchers investigating complex genetic traits, particularly diseases, by making it easy to investigate the hypotheses generated when examining systems with multiple perturbations. Usage of this web application requires no knowledge of programing or how to search relational databases. The data are easily accessed via a graphical user interface that retrieves pre-computed tables of genetic relationships and correlations. The database also contains detailed protocols and references for these studies.

**Figure 4:**
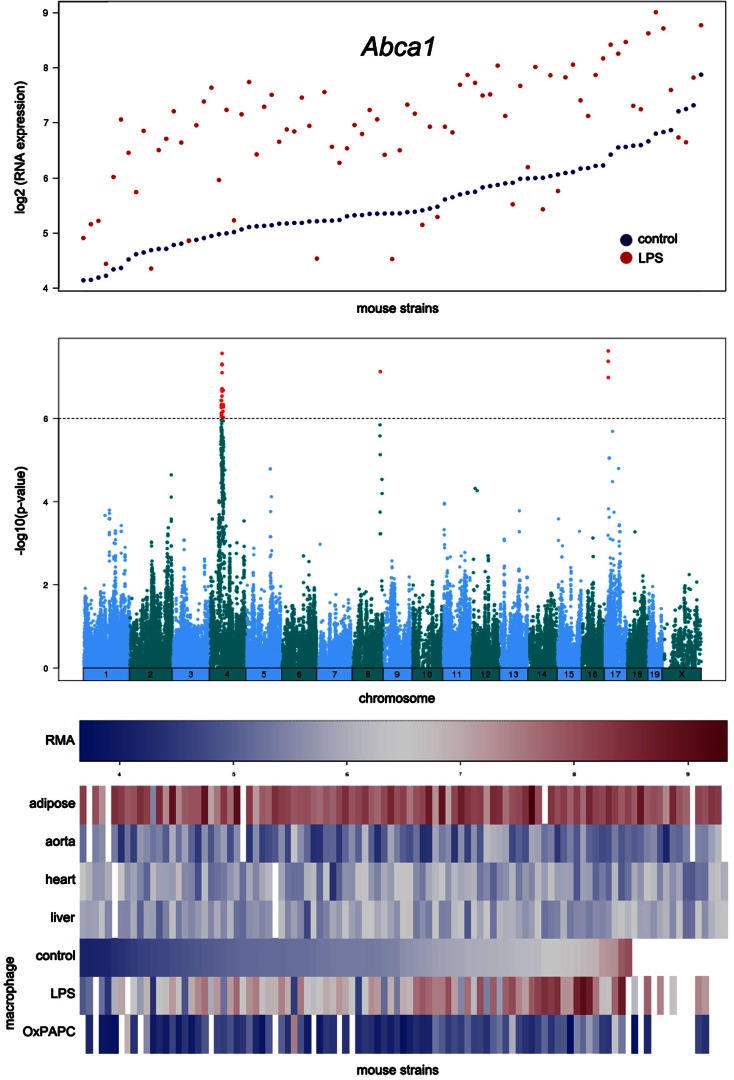
**Examples of data output from the SGR database**. This shows typical output for gene expression data in the HMDP. The example shown here for is Abca1, a gene involved in cholesterol efflux from cells. The top panel shows expression levels of the gene in macrophages cultured in the absence or presence of LPS. The middle panel shows the association analysis for Abca1 expression levels, graphing the genomic position of SNPs (*x*-axis) against their −log_10_ (*p*-value). The bottom panel shows a heat map for the expression of Abca1 in various tissues with expression levels ranging from very low (blue) to very high (red). [Figure from Orozco et al. (2012) with permission].

An example of the output from the database is shown in Figure 4. In this case, transcript levels of the gene for the Abca1 cholesterol transporter in macrophages are shown across the HMDP mouse panel. The upper panel shows transcript levels under two conditions, the middle panel shows loci controlling transcript levels, and the bottom panel shows expression in a variety of tissues as a heat map.

## Experimental Design of Data Sets

There are several types of data sets in the SGR. In this section, we briefly discuss each of these and summarize their applications, strengths, and limitations.

### Human population association studies

Cells or tissues from populations of unrelated human individuals were genotyped using high-density SNP arrays, and phenotyped at intermediate and clinical traits. There are two extensive human datasets in the database at present. The first consists of data derived from cultured endothelial cells from approximately 147 aortas obtained from heart transplant donors at UCLA (Romanoski et al., 2010, 2011). The primary clinical trait studied was the response to treatment of the endothelial cells with oxidized phospholipids, thought to be important in the development of atherosclerosis. The second resource is a collection of individuals studied by Markku Laakso in Kuopio, Finland, termed the METSIM study for METabolic Syndrome In Man (Stancáková et al., 2012). Altogether, this dataset contains over 10,000 individuals examined for many different metabolic traits. In addition, adipose biopsies have been obtained from about 1,200 of the individuals and expression arrays have been used to study transcript levels (both mRNA and microRNA) in adipose from a subset of these individuals. These latter data as of yet are unpublished and, therefore, access is restricted at present but will be publically available soon.

### Genetic crosses between inbred strains of mice

Systems genetics resource data sets in this category include several intercrosses between various inbred strains of mice (Table 1) that have been typed for cardiovascular and metabolic traits as well as transcript levels in one or more tissues. The data contain loci contributing to expression levels of thousands of transcripts and dozens of clinical traits. This experimental design has excellent mapping power but relatively poor resolution. Thus, significant genetic regions identified using this design tend to be many megabases in size and often contain hundreds of genes.

**Table 1:** **Data sets contained in the systems genetics resource**.

Study population	Clinical traits	Intermediate phenotypes	Reference
Hybrid mouse diversity panel: original panel analysis: chow fed, males, 16 weeks	Plasma lipids, bone density, body fat	Transcript levels (liver, heart)	Bennett et al. (2010), Suwanwela et al. (2011), Park et al. (2011), Ghazalpour et al. (2011), Farber et al. (2011)
Hybrid mouse diversity panel: follow up: chow fed, males, 16 weeks II	Plasma lipids, bone density, body fat		Bennett et al. (2010)
Hybrid mouse diversity panel: peritoneal macrophages, inflammatory responses	Response to oxidized phospholipids and LPS	Transcript levels	Orozco et al. (2012)
Metabolic syndrome in men (METSIM) study	Many metabolic and clinical traits	Adipose transcript levels, blood metabolites, proteins	Stancáková et al. (2012)
Human aortic endothelial cells [EC] culture	Response to oxidized phospholipids	Transcript levels	Romanoski et al. (2010, 2011), Gargalovic et al. (2006)
C57BL/6J × DBA/2J F2 on db/db background	Plasma lipids	Transcript levels (liver, adipose, duodenum)	Davis et al. (2012)
CeH/HeJ.Apoe−/− × C57BL/6JApoe −/− F2	Plasma lipids, body fat, atherosclerosis	Transcript levels (liver, adipose, brain, muscle)	Wang et al. (2006, 2007a,b), Yang et al. (2006), Chen et al. (2008), van Nas et al. (2009)
C3H/HeJ × C57BL/6J F2	Plasma lipids, body fat	Transcript levels (liver, adipose, brain, muscle)	van Nas et al. (2010), Farber et al. (2009), Orozco et al. (2009)
C57BL/6J × CAST/Ei F2	Plasma lipids, body fat	Transcript levels (liver, adipose, brain, muscle)	Langfelder et al. (2012)
C57BL/6J × DBA/2J F2	Plasma lipids	Transcript levels (liver)	Schadt et al. (2003), Colinayo et al. (2003)
Genome tagged mice, global congenic strains: C57BL/6J.DBA/2j and C57BL/6J.CAST/Ei	Many metabolic and clinical traits	NA	Davis et al. (2005), Terra et al. (2011)

### Outbred mouse stocks

Greatly improved mapping resolution can be obtained by examining outbred stocks using association analysis rather than linkage. The SGR contains one such study in which about 100 outbred mice were phenotyped for various metabolic traits and transcript levels in the liver, and genotyped for several thousand markers (Ghazalpour et al., 2008). Compared to linkage studies (above) the resolution is greatly improved. However, since only 100 individuals were examined, power in this study is quite low. This dataset is currently being added to the database.

### The hybrid mouse diversity panel

Over the last century, hundreds of inbred strains of mice have been produced by generations of brother-sister mating. However, most of these strains are genetically closely related, and only about 30–40 of the classic inbred strains are suitable for association analysis. Early association studies that used the classic inbred lines were flawed by their failure to deal with population structure. Also, 30 of these strains do not provide sufficient power for the analysis of most complex traits. An extension of the classical inbred strain design is the HMDP (Bennett et al., 2010) that increases the statistical power of classical association studies by including in the mapping panel a set of 70 recombinant inbred strains. In this design approximately 100 strains are phenotyped (30 classical inbred strains and 70 recombinant inbred strains) and association is performed after correcting for population structure (Kang et al., 2008). The mapping resolution with the HMDP is at least in order of magnitude better than using linkage, although not as good as human population association studies (Davis et al., 2013; Parks et al., 2013). Power is not as high as some linkage strategies, but there is about 80% power to identify loci that contribute to 5% of the total variance, which is adequate to capture at least a subset of the variations contributing to many complex traits (Bennett et al., 2010). An important advantage of the HMDP is that about 90% of the strains have been fully sequenced and the remainder typed using high-density SNP arrays. Another important strength of the HDMP data sets is that since the strains are inbred the data collected in each study is cumulative and can be applied to future studies.

### New datasets

Our website focuses primarily on data that includes: (1) genotypes, (2) intermediate phenotypes (such as whole genome transcript levels), and (3) clinical phenotypes (Table 2). We are particularly interested in data derived from the HMDP to allow integration across multiple phenotypes. Data is not screened for quality other than manuscript review for publication. At the moment, we do not have resources to bring in other publically available large datasets.

**Table 2:** **Summary of clinical phenotypes and intermediate phenotypes contained in the database**.

Data set	No. of microsatellites	No. of SNPs	No. of expression probes	No. of phenotypes
C57BL/6J × DBA/2J F2	130	NA	23,539	25
C3H/HeJApoE^−/−^ × C57 BL/6JApoE^−/−^ F2	NA	1,307	23,568	32
C3H/HeJ × C57BL/6J F2	NA	1,441	23,574	82
Hybrid mouse diversity panel (HMDP) (liver, adipose, heart, macrophage)	NA	132,285	22,416	197
Hybrid mouse diversity panel (HMDP) (bone)	NA	132,285	46,632	197
Human aortic endothelial cells	NA	934,968	18,630	NA
Metabolic syndrome in men (METSIM)	NA	640,492	16,223	

## Database Organization

**Figure 5:**
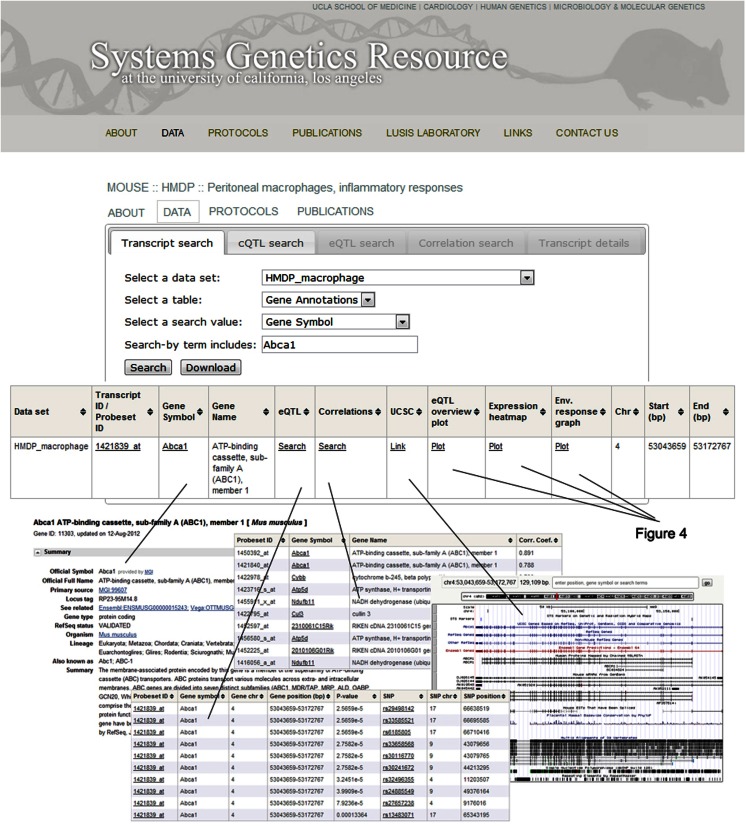
**Organization of the SGR database**. After selecting the *Abca1* gene in the HMDP macrophages dataset, a window will pop up with several options to help guide the researcher through the available SGR data and general information for that particular gene. For example, clicking on the “Gene symbol” tab links to NCBI (http://www.ncbi.nlm.nih.gov/) and the “UCSC” tab links to the UCSC genome browser (http://genome.ucsc.edu/). These sites provide various functional details on the gene as well as its location. The “correlations” tab produces a list of genes correlated with *Abca1*, including their gene symbol and the correlation coefficient. The “eQTL” tab provides a list of Abca1 eQTLs, including both detailed transcript and SNP information. Lastly, eQTL localizations, gene expression levels, and treatment response values are graphed by clicking on the “eQTL overview plot,” “Expression heatmap,” and “Environmental response graph” links, respectively.

The overall organization of the SGR database is shown in Figure 5. The primary data includes genotypes and intermediate and clinical phenotypes. A summary of the data is included under the home page. The derived data includes correlations among intermediate traits, correlations among clinical traits, correlations between intermediate and clinical traits, mapped loci for intermediate traits, and mapped loci for clinical traits.

### Navigation and usage

The main navigation menu provides options for looking at four aspects of each study. First, there is a general description that includes the original design goals and rationale. Second, there are detailed experimental protocols describing every procedure. Third, the data from each study can be explored at length and queried in multiple ways, with downloadable result tables and summary graphs. For example, after entering a gene symbol and clicking “Search” (Figure 5), clicking on the “Gene symbol” link in the resulting table takes one to the corresponding NCBI^3^ page, and the “UCSC” link takes one to the relevant genomic region on the UCSC genome browser^4^. It is also possible to search for significant associations and linkages using the “eQTL” link, as well as correlations by clicking the “Correlations” link. Gene expression levels, eQTL localizations, and treatment response values are graphed using the “Expression heatmap,” “eQTL overview plot,” and “Environmental response graph” links, respectively. Finally, the fourth available feature of each study is the lists of the resulting publications.

To give an overview of the ease of use of the SGR, we list here a few of the typical investigations that can be accomplished with the SGR, and the steps entailed. Several more detailed examples of applications that can be accomplished using the SGR are described in the following section.

Finding expression QTLs:

1. Choose one of the datasets marked as “publicly available” under the Data menu.
2. Enter a gene symbol to search for and click Search.
3. From the resulting list of probes, click the “search” link in the eQTL column.
4. If applicable, choose a treatment condition.
5. Choose a significance cutoff and click Find.

Finding correlations between different genes:

1. Choose one of the datasets marked as “publicly available” under the Data menu.
2. Enter a gene symbol to search for and click Search.
3. From the resulting list of probes, click the “search” link in the Correlations column.
4. If applicable, choose a treatment condition.
5. Choose a significance cutoff and click Find.

Finding correlations between genes and traits:

1. Choose one of the datasets marked as “publicly available” under the Data menu.
2. Enter a gene symbol to search for and click Search.
3. From the resulting list of probes, click the “search” link in the Correlations column.
4. If applicable, choose a treatment condition.
5. Select “Trait” from the dropdown that says Gene if it is available.
6. Choose a significance cutoff and click Find.

### Quality control

The publically available data have undergone review during publication but there is no additional quality control. We provide false discovery rates or *p*-values for the relationships analyzed but it is up to the investigators to determine the level of significance desired. Thresholds for significance for correlations and mapping results can be found in the associated publications. An important use of the database is hypothesis generation, which requires a reduced level of significance.

### Relationship mining

The data consist of genotypes, intermediate phenotypes, and clinical traits. Data can be mined by testing for correlations between the intermediate phenotypes and clinical traits, between different intermediate phenotypes, and between different clinical traits. Also, the locations of genes perturbing the intermediate phenotypes (such as eQTL) and clinical traits can be downloaded to test for co-mapping or other relationships. Causality testing can be performed as described in Schadt et al. (2005) and Aten et al. (2008). Expression data can be used to model biologic networks (Langfelder et al., 2012). At present, we have not incorporated meta-analysis of the data but this has been performed for a subset of the data in van Nas et al. (2010) and Furlotte et al. (2012). Examples of data mining are provided below.

### Technical details

The SGR comprises a web interface written using PHP, XHTML, and CSS. Images are generated dynamically on the Linux web server using PHP and R, and client-side interactivity takes advantage of the jQuery 1.4+ JavaScript library. The data is structured in multiple, read-only databases hosted on Microsoft SQL Server 2008. The web and database servers are separate, dedicated machines. The only public access to the database server is via the web server.

### Resource availability

All published results are freely available, no login required. Access to unpublished data requires an account that can be obtained through the site administrator after consultation and agreement with the relevant investigator.

## Examples of Database Applications

In this section, we describe several additional applications of the SGR. In some cases, the analysis can be retrieved immediately from the pre-computed linkage and association results or tables of correlations. In other cases, such as the modeling of gene networks or analysis of enrichment for literature-based categories (such as Gene Ontology) the data must be downloaded from the SGR and then further analyzed with an additional software package.

**Figure 6:**
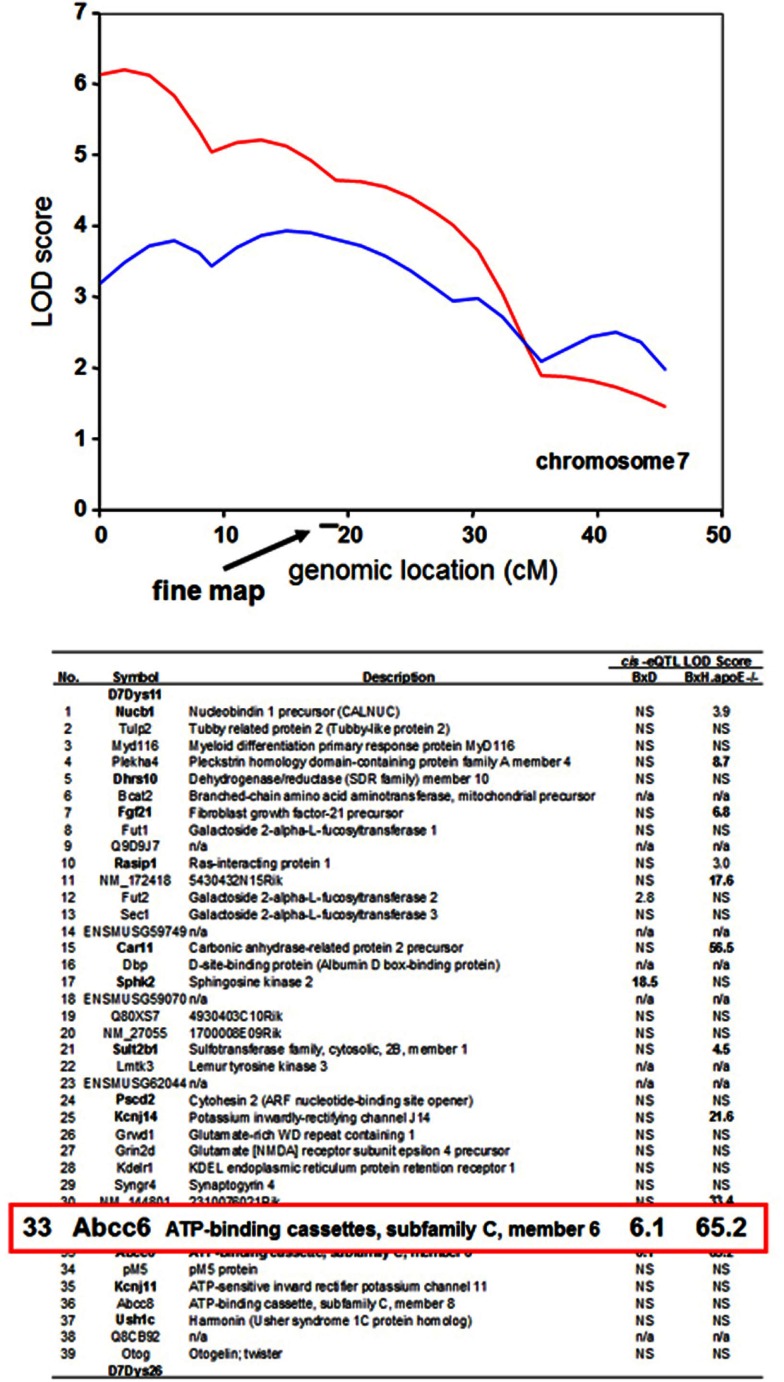
**Use of expression quantitative trait loci (eQTL) to prioritize candidate genes at a locus contributing to a complex trait**. The trait of dystrophic cardiac calcinosis was mapped by linkage analysis in two mouse F2 intercrosses (BxD and BxH.apoe^−/−^). The peak region in the two crosses contains a total of 39 genes. Expression array analysis on liver samples was carried out on mice, resulting in the mapping of several thousand *cis*-eQTL loci. The genes exhibiting *cis*-eQTL at the dystrophic cardiac calcinosis locus on chromosome 7 are shown according to their LOD scores. The *Abcc6* transporter was the only gene exhibiting a significant *cis*-eQTL activity in both crosses. Genes exhibiting non-significant LOD scores are indicated by “NS,” and genes not on the arrays are indicated by “n/a.” [Figure based on data from Meng et al. (2007)].

One straightforward application is *to* *prioritize candidate genes at a locus contributing to a complex trait*. For example, a locus determining dystrophic cardiac calcification (DCC) was mapped to a region of chromosome 7 using linkage analysis in a cross between the strains DBA/2 and C57BL/6 (Ivandic et al., 1996). It was subsequently mapped in a second cross between C3H/HeJ and C57BL/6J to the same region (Meng et al., 2007). Global transcript levels had been measured in the livers of mice from both crosses, and genes exhibiting *cis*-regulation of transcript levels (*cis* eQTLs) were identified. All this data is available in the database. The SGR shows that the *Abcc6* gene encoding an orphan ABC transporter exhibits a strong *cis*-eQTL effect in both crosses, whereas the vast majority of the genes at the locus show no evidence of *cis*-eQTL regulation (Ivandic et al., 1996). Thus, the Abcc6 gene would be a priority candidate for this trait. In fact, the *Abcc6* gene was shown to underlie the dystrophic cardiac calcinosis using transgenic complementation (Meng et al., 2007) (Figure 6).

Another application of the SGR is *to* *identify pathways through which a gene mutation contributes to a complex phenotype*. One can interrogate the database for molecular phenotypes (such as transcript levels) that are perturbed by a particular gene mutation. One can then ask if these sets of phenotypes are enriched for any known pathways. For example, it remains unknown how the *Abcc6* gene contributes to vascular and cardiac calcification in mice (and a similar phenotype in humans seen in the *pseudoxanthoma* *elasticum* disorder). Evidence had been obtained that the transporter was present in the cytoplasmic membrane of liver hepatocytes, suggesting that it transported an unknown substance out of hepatocytes into the circulation and that the absence of this substance promoted vascular calcification. However, when Martin et al. (2012), using the HMDP data now in SGR, examined the liver transcripts associated with *Abcc6* deficiency, they observed that the most strongly associated genes were correlated in Gene Ontology with mitochondrial functions (Table 3). They followed up by carrying out detailed biochemical analysis of localization of the *Abcc6* protein and showed that it, indeed, is localized to the mitochondria-associated membrane rather than the plasma membrane (Martin et al., 2012).

**Table 3:** **Identification of pathway underlying complex traits using gene enrichment**.

Gene ontology cellular localization	Fold enriched	Fisher’s exact test	*p*-Value
Mitochondrion (GO:005739)	2.0	2.8 × 10^−9^	5.9 × 10^−9^
Mitochondrial part (GO:0044429)	2.2	2.3 × 10^−5^	5.2 × 10^−5^
Golgi apparatus (GO:0005794)	1.9	8.0 × 10^−5^	1.6 × 10^−4^
Mitochondrial envelope (GO:0005740)	2.1	4.4 × 10^−4^	1.0 × 10^−3^
Endosome (GO:0005768)	2.5	4.0 × 10^−4^	1.0 × 10^−3^

*Numerous strains in the HMDP exhibit a null mutation for the *Abcc6* gene that contributes to dystrophic cardiac calcinosis. A list of genes strongly associated with the *Abcc6* null mutation in the HMDP is highly enriched with mitochondrial functions in the Gene Ontology pathways. This suggests that *Abcc6* may reside in the mitochondria rather than in the cytoplasmic membrane as previously thought. Martin et al. (2012) proved this alternative localization to be correct*.

**Figure 7:**
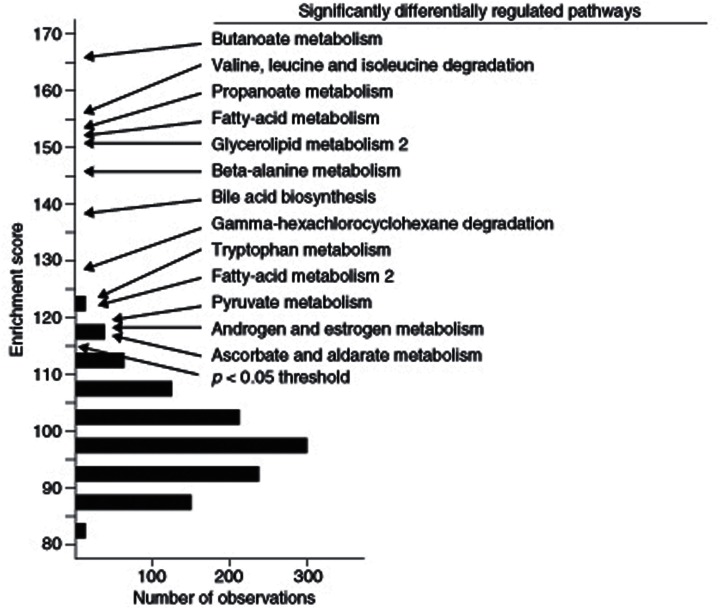
**Identification of literature-based pathways contributing to complex traits using gene enrichment analysis**. In this study, the F2 intercross between strains C57BL/6J and DBA/2J were studied for adiposity. Expression arrays were carried out on liver RNA and genes that are differentially expressed between lean and obese mice were identified. KEGG pathways enriched in the differentially expressed genes were then identified. Those exceeding a *p*-value of 0.05 in the enrichment score are indicated. [Figure from Ghazalpour et al. (2005) with permission].

The enrichment analysis results found in the SGR can also be used *to* *identify pathways associated with a particular complex trait rather than variation of a specific gene*. For example, Ghazalpour et al. (2005) analyzed a segregating population of mice from an intercross between C57BL/6J and DBA/2J that were studied for obesity related traits and for global gene expression in liver. They asked which literature-based pathways were significantly associated with obesity traits using Gene Set Enrichment Analysis (GSEA) as well as another enrichment approach. They observed that 13 KEGG pathways, centered on the tricarboxylic acid cycle, were significantly enriched (Ghazalpour et al., 2005) (Figure 7). Subsequent transgenic experiments validated a number of the pathways in genes predicted using this enrichment analysis (Yang et al., 2009).

**Figure 8:**
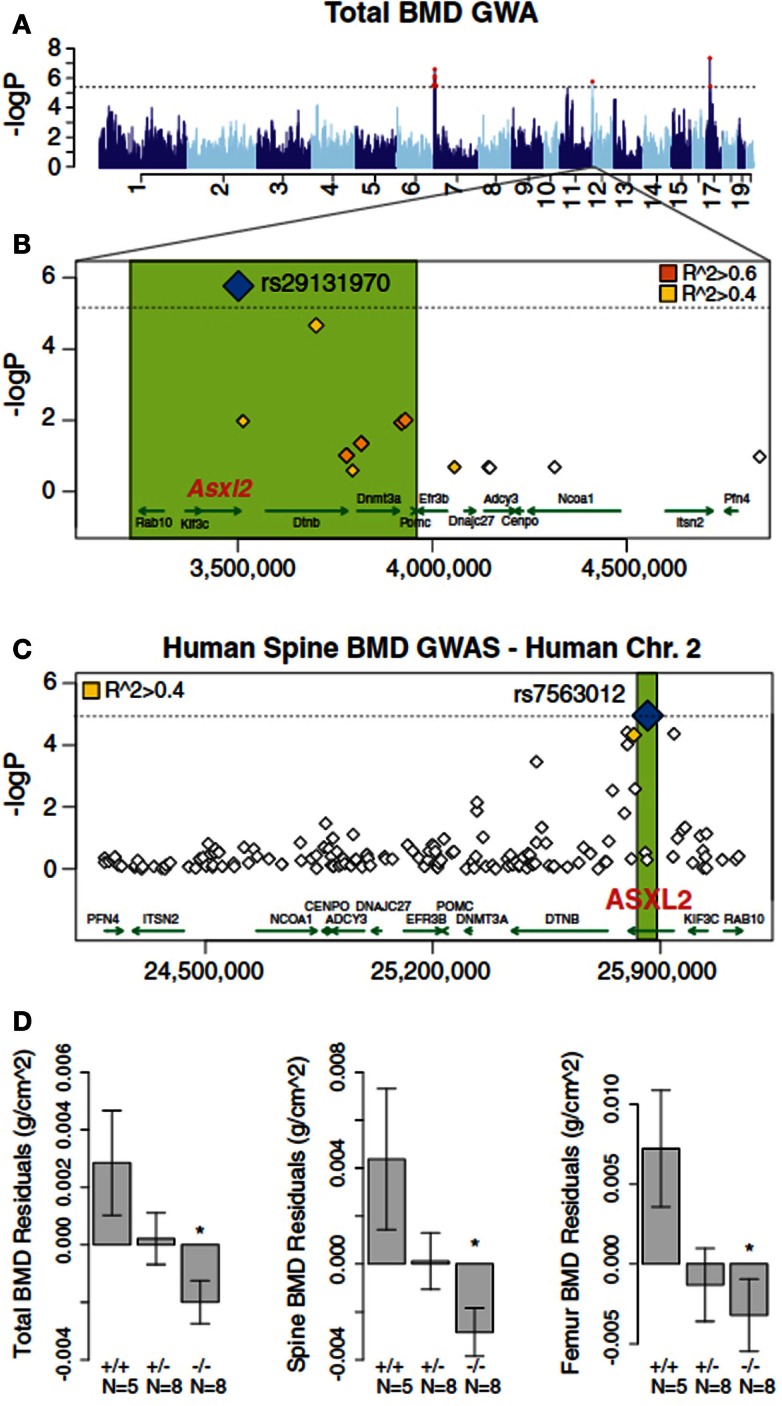
**Mapping complex clinical traits using the HMDP data in the SGR**. Variation in Asxl2 in mice and humans is associated with bone mineral density (BMD). **(A)** A genome-wide association study in the HMDP for total BMD identified a significant region on chromosome 12. **(B)** A non-synonymous SNP (rs29131970) in the *Asxl2* gene that was predicted to alter protein function was the most significantly associated chromosome 12 SNP in the HMDP. **(C)** Human SNPs within ASXL2 were also associated with BMD in roughly 6,000 Icelandic individuals. **(D)** Male mice deficient in Asxl2 (−/−) display significant decreases relative to wild-type controls in total BMD, spine BMD, and femur BMD residuals after adjusted for age and body weight. Data shown in **(D)** are residual mean ± SEM.

An example of the use of the HMDP data in the SGR *to* *carry out high resolution mapping of complex traits* is a study by Farber et al. (2011). In this study, bone density was measured in each of the roughly 100 strains of the HMDP and association analysis was carried out using the EMMA algorithm (Kang et al., 2008) to correct for population structure. Significant association was observed on chromosome 19 and the locus contained a single novel gene, *Asxl2*. The identity of the gene as a regulator of bone mineral density and osteoclastogenesis was shown using a knockout model (Farber et al., 2011). Furthermore, the same gene exhibited suggestive, although not significant, association with bone marrow density in a human population study (Farber et al., 2011) (Figure 8).

**Figure 9:**
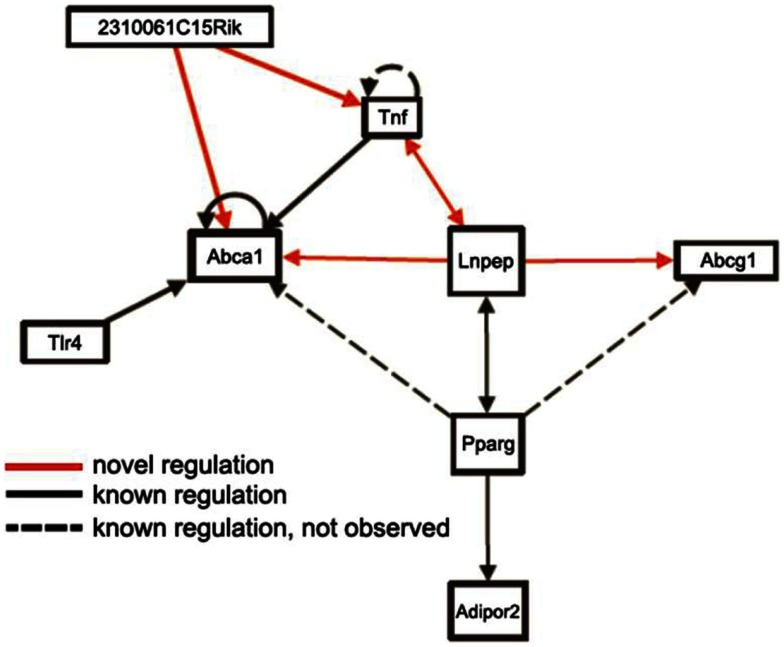
**Use of *trans*-eQTLs to identify novel gene-gene interactions**. The expression data for macrophages in the HMDP data in the SGR were used to identify *cis-* and *trans-*acting eQTL genome-wide. Based on this, genes likely to contribute to the expression of other genes in *trans* were identified. This suggested a number of novel regulatory pathways (arrows shown in red) as well as known regulatory pathways (shown in solid lines). Some known regulatory pathways (dotted lines) were not observed, which is not unexpected since not all genes will exhibit common variation in the population. [Figure from Orozco et al. (2012) with permission].

Mapping loci controlling intermediate phenotypes, such as transcript levels, protein levels, and metabolite levels, provides a potentially powerful approach *to identify novel interactions and pathways*. In particular, *trans*-acting eQTLs can relate genetic variations in one gene to changes in the expression of a distal gene. For example, Romanoski et al. (2011) used this approach and data in the SGR to identify the G protein coupled receptor GPR39 as a model regulator of heme oxygenase in human endothelial cells. Similarly, Orozco et al. (2012) used eQTLs in peritoneal macrophages isolated from mice to identify novel interactions influencing the expression of the Abca1 cholesterol transporter (Figure 9).

**Figure 10:**
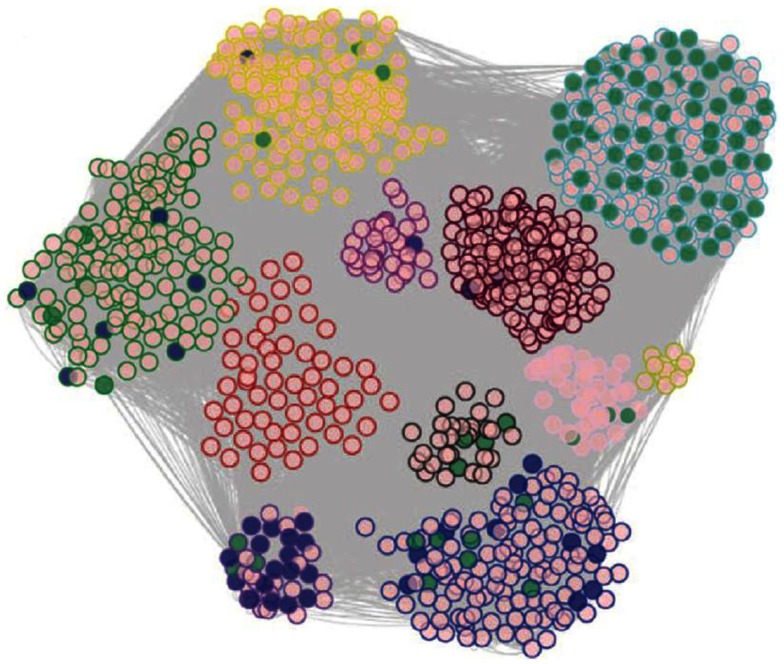
**Modeling of biologic networks using co-expression analysis**. In this study, cultured endothelial cells from roughly 147 human subjects were analyzed using expression arrays before and after treatment of oxidized phospholipids. A set of about 2,000 genes that were regulated by the oxidized phospholipids were selected and analyzed using co-expression network analysis using the WGCNA algorithm, developed by Horvath and colleagues (labs.genetics.ucla.edu/horvath/CoexpressionNetwork). A total of 11 modules of tightly correlated genes were observed in the network and most of these were highly enriched for certain Gene Ontology pathways. [Figure from Romanoski et al. (2010) and Romanoski et al. (2011) with permission].

The intermediate phenotype data in the SGR have also proven very useful *to* *model biologic networks through methods such as co-expression analysis*. For example, Romanoski et al. (2011) examined endothelial cells isolated from about 147 human subjects in culture. They examined gene expression patterns before and after treatment with oxidized phospholipids, which are thought to contribute to the chronic inflammation in atherosclerosis and perhaps other chronic inflammatory diseases. They used genes that were perturbed by oxidized phospholipids to construct a biological co-expression network (Figure 10). The individual modules of the network were highly enriched for Gene Ontology categories and helped reveal novel pathways contributing to vascular inflammation (Romanoski et al., 2011).

## Discussion

We are dedicated to continuing to update the SGR both with each new available study and by expanding the software’s features and ease of use. For example, the next software update will allow one to search for associations for a particular SNP across different data sets. We plan on expanding the database by adding roughly five new data sets each year.

By making available the user-friendly SGR, we hope to encourage the genetics community to mine the vast storehouse of complex trait genetics data that continues to accumulate. We have no doubt that many discoveries are waiting to be found. We welcome any suggestions for improving this web application and making it even more useful to the community.

## Conflict of Interest Statement

The authors declare that the research was conducted in the absence of any commercial or financial relationships that could be construed as a potential conflict of interest.

## Footnotes

^1^www.genenetwork.org

^2^www.phenome.jax.org

^3^http://www.ncbi.nlm.nih.gov/

^4^http://genome.ucsc.edu/
